# Associations Between Nutrient Intake and Vascular Inflammation Among Healthy Adults Living in Rural and Peri-Urban Particulate Matter 2.5-Affected Areas: An Exploratory Study

**DOI:** 10.3390/nu17172867

**Published:** 2025-09-04

**Authors:** Wason Parklak, Hataichanok Chuljerm, Sawaeng Kawichai, Puriwat Fakfum, Putita Jiraya, Praporn Kijkuokool, Wiritphon Khiaolaongam, Surasawadee Somnuk, Kanokwan Kulprachakarn

**Affiliations:** 1 Research Center for Non-Infectious Diseases and Environmental Health, Research Institute for Health Sciences, Chiang Mai University, Chiang Mai 50200, Thailand; wason.p@cmu.ac.th (W.P.); hataichanok.ch@cmu.ac.th (H.C.); sawaeng.kaw@cmu.ac.th (S.K.); nuengpuriwat@gmail.com (P.F.); putita_jiraya@cmu.ac.th (P.J.); 2 School of Health Sciences Research, Research Institute for Health Sciences, Chiang Mai University, Chiang Mai 50200, Thailand; praporn_k@cmu.ac.th (P.K.); wiritphon_k@cmu.ac.th (W.K.); 3 Department of Sports Science, Faculty of Sports and Health Science, Kasetsart University, Kamphaeng Saen Campus, Nakhon Pathom 73140, Thailand; surasawadee.so@ku.th

**Keywords:** cardiovascular risk, nutrient intake, particulate matter 2.5, peri-urban area, rural area, vascular inflammation

## Abstract

**Background/Objectives:** Chronic particulate matter 2.5 (PM2.5) exposure is associated with vascular inflammation and cardiovascular risk. However, the role of diet in modulating inflammation under such conditions remains unclear. This study explored the associations between nutrient intake and circulating vascular inflammatory biomarkers among apparently healthy adults living in PM2.5-affected rural and peri-urban areas in Chiang Mai Province, Thailand. **Methods:** Fifty-three healthy adults (27 rural; 26 peri-urban) were assessed for sociodemographic characteristics, clinical parameters, and dietary intake using three consecutive 24 h recalls. Serum levels of intercellular adhesion molecule-1 (ICAM-1), vascular cell adhesion molecule-1 (VCAM-1), and interleukin-6 (IL-6) were measured. Multiple linear regression was used to analyze associations between nutrient intake and inflammatory markers, adjusting for potential confounders. **Results:** Peri-urban participants exhibited significantly higher levels of ICAM-1, VCAM-1, and IL-6 compared to rural participants (*p* < 0.05). They also had higher intakes of sugars and saturated fatty acids, whereas rural participants consumed more cholesterol, antioxidant nutrients (vitamins C, A, and E), and minerals (e.g., potassium, selenium). Regression analyses revealed positive associations between sugar intake and all three inflammatory markers (ICAM-1: *β* = 0.467; VCAM-1: *β* = 0.481; IL-6: *β* = 0.557; all *p* ≤ 0.001). In contrast, intakes of selenium and vitamin A were inversely associated with VCAM-1 levels. **Conclusions:** These findings suggest that certain dietary components may influence vascular inflammation among individuals exposed to PM2.5. Encouraging consumption of anti-inflammatory nutrients may help mitigate pollution-related cardiovascular risks.

## 1. Introduction

Ambient fine particulate matter (PM2.5) is a well-established environmental risk factor for cardiovascular disease (CVD), primarily through mechanisms involving vascular injury and systemic inflammation [1]. Due to its small aerodynamic diameter (<2.5 µm), PM2.5 can bypass upper respiratory defenses, penetrate the alveoli, and enter the bloodstream, where it exerts harmful effects on vascular endothelium [2]. A key mechanistic link between PM2.5 exposure and CVD is vascular inflammation, which precedes endothelial dysfunction, arterial stiffening, and atherosclerotic plaque development. At the molecular level, PM2.5 stimulates excessive generation of reactive oxygen species (ROS), initiating oxidative stress, mitochondrial dysfunction, and endoplasmic reticulum stress [3]. These redox imbalances activate pro-inflammatory signaling cascades such as nuclear factor-kappa B (NF-κB), mitogen-activated protein kinases (MAPKs), and the NOD-like receptor family pyrin domain-containing 3 (NLRP3) inflammasome, leading to upregulation of cytokines [interleukin (IL)-6, tumor necrosis factor-alpha (TNF-α), IL-1β] and adhesion molecules [1]. At the cellular level, these responses disrupt endothelial integrity, enhance vascular permeability, and promote leukocyte adhesion, thereby amplifying systemic inflammation. In parallel, vascular smooth muscle cells undergo phenotypic switching and proliferation, while macrophages polarize toward a pro-inflammatory state, all of which accelerate vascular remodeling and calcification [3]. Collectively, these processes establish a biological pathway by which PM2.5 exposure drives vascular inflammation and elevates the risk of CVD, including hypertension, atherosclerosis, myocardial infarction, and stroke [1,2,3,4].

Key biomarkers of PM2.5-induced vascular inflammation include IL-6, intercellular adhesion molecule-1 (ICAM-1), and vascular cell adhesion molecule-1 (VCAM-1) [4]. IL-6, secreted by macrophages, endothelial cells, and adipocytes, enhances immune responses and stimulates hepatic production of acute-phase proteins. Elevated IL-6 levels are linked to increased cardiovascular risk and atherosclerosis progression [5,6]. ICAM-1 and VCAM-1, expressed on activated endothelial cells, facilitate leukocyte adhesion and transmigration [7]. PM2.5 exposure upregulates these molecules through redox-sensitive signaling, contributing to endothelial dysfunction and chronic vascular inflammation [8]. Although non-specific, C-reactive protein (CRP), a downstream marker of IL-6 activity, is also elevated in response to PM2.5 and reflects systemic inflammation [9]. Meta-analysis studies show a dose–response relationship between PM2.5 exposure and increased levels of these biomarkers [8,10]. Furthermore, vascular inflammation not only plays a central role in atherogenesis but also contributes to thrombotic complications, plaque instability, and endothelial dysfunction—all hallmarks of PM2.5-mediated cardiovascular toxicity [11].

However, evidence indicates that certain dietary patterns can modulate systemic inflammation and lower CVD risk, even in the presence of environmental stressors like air pollution [12,13]. Diets abundant in fruits, vegetables, whole grains, legumes, and unsaturated fats—such as the Dietary Approaches to Stop Hypertension (DASH), Mediterranean, and traditional rural diets—are associated with reduced levels of inflammatory biomarkers, including IL-6, TNF-α, CRP, and adhesion molecules [14,15]. These diets provide essential micronutrients, antioxidants, and phytochemicals that combat oxidative stress and endothelial dysfunction caused by pollutants [16]. In contrast, Western dietary patterns—characterized by high intakes of refined carbohydrates, added sugars, saturated and trans fats, and processed foods—have been linked to elevated vascular inflammation and higher cardiometabolic risk [17,18,19]. This shift toward unhealthy eating is becoming more common globally, including in Southeast Asia, due to urbanization, economic transitions, and changing food systems. Traditional diets in peri-urban and urban settings are being replaced by energy-dense, nutrient-poor alternatives, contributing to increased rates of chronic diseases and inflammation-related outcomes [17,19].

Chiang Mai, a northern Thai province, experiences seasonal PM2.5 pollution—particularly during February to April—resulting in hazardous air quality and increased cardiopulmonary risks. Chronic exposure to PM2.5 is linked to oxidative stress, inflammation, and heightened CVD risk [20,21,22]. Despite this environmental burden, lifestyle and dietary habits differ between rural and peri-urban residents. Rural communities often maintain traditional diets and active lifestyles, whereas peri-urban populations tend toward more sedentary behavior and modern dietary patterns. This study aimed to examine the association between nutrient intake and vascular inflammation among healthy adults living in PM2.5-affected rural and peri-urban areas of Chiang Mai. Understanding the interaction between diet quality and environmental exposures is critical for developing public health strategies and targeted nutritional interventions to reduce cardiovascular risk in vulnerable populations.

## 2. Materials and Methods

### 2.1. Study Locations

**Figure 1:**
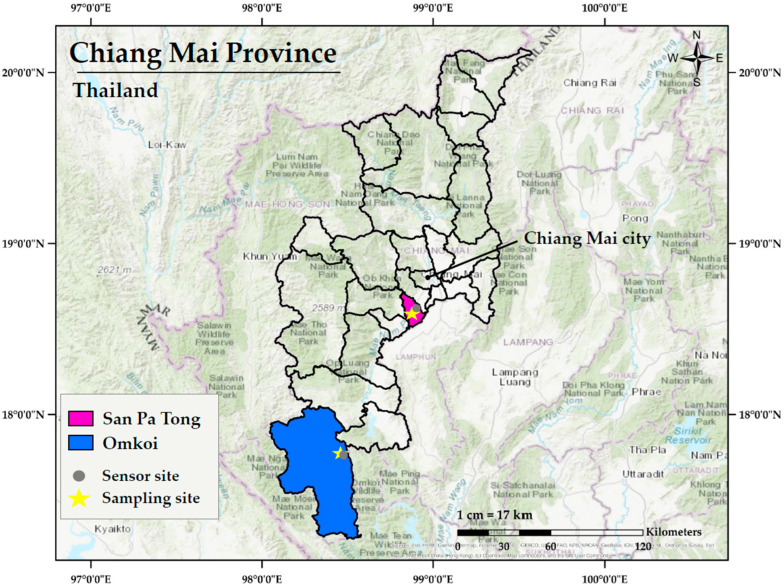
Study sites in Chiang Mai Province, Thailand, indicating locations of ambient PM2.5 monitoring stations and participant recruitment sites in rural (Omkoi District) and peri-urban (San Pa Tong District) areas.

The geographic locations of the study areas and data collection sites are presented in Figure 1. This study was conducted in two districts of Chiang Mai Province, located in Northern Thailand: Omkoi District, a predominantly rural area characterized by forested and mountainous terrain, situated approximately 200 km southwest of Chiang Mai city; and San Pa Tong District, a peri-urban area located about 35 km southwest of the city. In Omkoi, the PM2.5 sensor and participant screening site were situated at the Ban Yang Piang Subdistrict Health Promoting Hospital in Yang Piang Subdistrict. In San Pa Tong, the PM2.5 sensor was installed at the San Pa Tong Subdistrict Municipality Office, while participant screening was carried out at the Ban Hua Rin Subdistrict Health Promoting Hospital in Thung Satok Subdistrict.

The PMS7003 sensors, which were manufactured by Beijing Plantower Co., Ltd. (Beijing, China), were employed to detect ambient PM2.5 concentrations. Based in Chiang Mai, Thailand, Nano-generation Co., Ltd. integrated these sensors into monitoring devices and calibrated them for humidity-related adjustments. Daily PM2.5 readings were collected and accessed via the Northern Thailand Air Quality and Health Index (NTAQHI) platform [https://www2.ntaqhi.info/ (accessed on 28 October 2024)], which is administered by the Research Institute for Health Sciences (RIHES), Chiang Mai University. The calibration procedures, data processing (including averaging), and quality assurance were conducted in accordance with the established protocols developed by RIHES. From data collected over a three-year period (2021–2023), Table 1 illustrates the annual average PM2.5 concentrations. The San Pa Tong District (peri-urban area) exhibited a mean concentration of 19.27 ± 7.32 µg/m^3^, while the Omkoi District (rural area) exhibited an annual mean of 15.58 ± 5.60 µg/m^3^. It is noteworthy that the annual average concentrations of PM2.5 in both districts exceeded the exposure limit of 5 µg/m^3^ set by the WHO guideline [23].

### 2.2. Ethical Considerations

The research protocol was submitted to the Human Experimentation Committee of the Research Institute for Health Sciences, Chiang Mai University, for ethical review on 19 December 2023. Ethical approval was subsequently granted on 29 May 2024, under reference number 30/2024. Prior to participation, all individuals received a detailed explanation of the study’s aims and procedures. Written informed consent was obtained from every participant before enrollment.

### 2.3. Study Subjects

Participants selected for this study were required to meet the following inclusion criteria:

- Aged 35 years or older;
- Had resided continuously in either Omkoi or San Pa Tong District for at least three years without relocation during that period;
- In generally good health;
- Free from any diagnosed serious medical conditions, including but not limited to cardiovascular disease, chronic kidney disease, liver disease, gout, cancer, or hereditary disorders such as thalassemia;
- Free from any ongoing infections at the time of enrollment;
- Not pregnant or breastfeeding;
- Not currently dependent on illicit substances or chronically addicted to alcohol;
- Without psychiatric disorders that could interfere with their ability to participate in the study.

Prior to data collection and the acquisition of biological samples, all participants were thoroughly informed about the objectives and procedures of the study.

### 2.4. Study Design

This exploratory study aimed to investigate differences in lifestyle behaviors—including smoking, alcohol consumption, dietary intake, and physical activity—and their associations with vascular inflammation markers among apparently healthy adults residing in rural and peri-urban areas affected by PM2.5 air pollution in Chiang Mai Province, Thailand. Data collection took place in June 2024 in two distinct locations: Omkoi District, representing a rural mountainous region, and San Pa Tong District, representing a peri-urban area. A structured questionnaire was administered to collect demographic and lifestyle information. Physical activity was assessed using the short-form International Physical Activity Questionnaire (IPAQ-SF), with total physical activity levels reported in the metabolic equivalent of task (MET)-minutes per week.

Anthropometric and physiological assessments included measurements of body weight, height, waist circumference (WC), hip circumference (HC), and blood pressure. Venous blood samples were obtained to evaluate fasting blood glucose (FBG), lipid profile, liver and kidney function, and serum electrolyte concentrations. All blood samples were processed and analyzed at the Clinical Service Center, Faculty of Associated Medical Sciences, Chiang Mai University. Serum was separated by centrifugation and stored at −80 °C for the subsequent analysis of vascular inflammation-related biomarkers.

### 2.5. Assessment of Serum Vascular Inflammatory Biomarkers

The serum levels of key vascular inflammatory biomarkers—intercellular adhesion molecule-1 (ICAM-1), vascular cell adhesion molecule-1 (VCAM-1), and interleukin-6 (IL-6)—were determined using enzyme-linked immunosorbent assay (ELISA) with colorimetric detection. Commercial ELISA pair sets were employed for each target analyte, specifically: Human ICAM-1 (Cat. No. SEKA10346), VCAM-1 (Cat. No. SEK10113), and IL-6 (Cat. No. SEKB10395), all supplied by Sino Biological Inc. (Wayne, PA, USA). Absorbance values were read at a wavelength of 450 nm using a microplate reader (BMG Labtech, Ortenberg, Germany). Each sample was analyzed in duplicate to ensure precision, and concentrations were derived from standard curves generated using recombinant protein standards. Final results were reported in either picograms per milliliter (pg/mL) or nanograms per milliliter (ng/mL), depending on the analyte.

### 2.6. Dietary Intake Assessment

Dietary intake was assessed using three consecutive 24 h dietary recall interviews, covering two weekdays and one weekend day. To reduce recall bias, participants were informed one week in advance and were encouraged to keep a record or take photographs of all foods and beverages consumed during the specified days. Throughout the data collection period, trained research personnel conducted in-person interviews using a standardized questionnaire. To enhance the accuracy of portion size estimation, visual aids such as plates, bowls, measuring cups, and spoons were employed. Nutrient and energy intakes were analyzed using INMUCAL Nutrients V.4.0 (Institute of Nutrition, Mahidol University, Nakhon Pathom, Thailand), a dietary assessment software. Daily intake data were computed and expressed as mean daily consumption per individual.

### 2.7. Statistical Analysis

Data analysis was conducted using SPSS Statistics version 15.0 (SPSS Inc., Chicago, IL, USA). Descriptive statistics were applied to characterize the study population. Continuous variables were summarized as means with standard deviations (mean ± standard deviation [SD]), while categorical variables were presented as frequencies (n) and percentages (%). Comparative analyses between participants from rural and peri-urban areas were carried out using the chi-square or Fisher’s exact tests for categorical variables, including sociodemographic information, lifestyle behaviors, and self-reported health conditions (e.g., sex, age categories, smoking and alcohol use, and chronic disease presence). Due to the non-normal distribution of continuous variables—such as anthropometric data, biochemical markers, and dietary intake—the Mann–Whitney U test was used for between-group comparisons. Statistical significance was set at a *p*-value of less than 0.05. To identify independent predictors of serum inflammatory biomarker concentrations, multiple linear regression analyses were performed. The findings were reported as standardized beta coefficients (*β*) along with their corresponding *p*-values. To account for multiple comparisons across nutrient–biomarker associations, the false discovery rate was controlled using the Benjamini–Hochberg (BH) procedure, with a significance level set at Q = 0.05. For each family of comparisons (m), two-sided *p*-values were ranked in ascending order and compared to their BH critical values (i/m) × Q. The largest *p*-value meeting this threshold defined the set of statistically significant results. BH-adjusted *p*-values were computed using the step-up procedure and reported alongside unadjusted values; associations with BH-adjusted *p* < 0.05 were considered statistically significant.

## 3. Results

### 3.1. Sociodemographic and Behavioral Characteristics of Participants

Sociodemographic and behavioral characteristics of participants residing in rural and peri-urban areas are presented in Table 2. A total of 53 apparently healthy individuals participated in the study, including 27 residents from the rural area (Omkoi District) and 26 from the peri-urban area (San Pa Tong District). Statistically significant differences in several demographic and lifestyle factors were observed between the two groups. The distribution of sex varied markedly between locations (*p* < 0.001), with a higher proportion of males in the rural group (48.15%) compared to the peri-urban group (3.85%). Participants in the rural area were also significantly younger than those in the peri-urban area (50.04 ± 7.58 vs. 56.04 ± 7.12 years, *p* = 0.005).

Regarding alcohol consumption, a higher percentage of rural participants reported having consumed alcohol (74.07%) compared to their peri-urban counterparts (38.46%), with this difference reaching statistical significance (*p* = 0.009). Smoking status also differed significantly (*p* = 0.002), with current smoking more prevalent in the rural group (33.33%), whereas no current smokers were reported in the peri-urban group.

Total physical activity levels were significantly higher among rural participants compared to those in peri-urban areas (9492.0 [2772.0–17,598.0] vs. 2451.0 [1339.5–8671.5] MET-min/week; *p* = 0.020). In particular, vigorous physical activity tended to be greater in the rural group (3360.0 [0.0–13,440.0] vs. 720.0 [0.0–4800.0] MET-min/week; *p* = 0.082), although the difference did not reach statistical significance. Conversely, moderate physical activity was significantly higher among peri-urban participants (1320.0 [360.0–1680.0] vs. 480.0 [0.0–720.0] MET-min/week; *p* < 0.001). Walking activity was also greater in the rural group (1584.0 [594.0–4158.0] vs. 297.0 [0.0–1386.0] MET-min/week; *p* = 0.019).

### 3.2. Physical Examination Parameters of Participants

The physical examination parameters of participants from rural and peri-urban areas are provided in Table 3. While no statistically significant differences were observed for body height, body weight, WC, waist-to-hip ratio (WHR), blood pressure, or heart rate between the two groups, a significant difference in body mass index (BMI) was noted. Participants in the peri-urban area exhibited a significantly higher BMI compared to those in the rural area (25.76 ± 3.76 vs. 23.33 ± 4.92 kg/m^2^, *p* = 0.004). Although WC tended to be higher among peri-urban participants (84.17 ± 9.54 cm) than their rural counterparts (79.94 ± 12.22 cm), this difference did not reach statistical significance (*p* = 0.058). Similarly, no significant differences were found in WHR when stratified by sex.

### 3.3. Clinical Biochemistry Profiles of Participants

Table 4 presents the clinical biochemistry profiles of participants from rural and peri-urban areas. Participants residing in peri-urban areas exhibited slightly higher fasting blood glucose (FBG) levels compared to rural participants (100.85 ± 14.03 vs. 97.19 ± 18.59 mg/dL), with the difference reaching statistical significance (*p* = 0.050). No significant differences were observed in lipid profiles, including total cholesterol (TC), triglycerides (TG), high-density lipoprotein cholesterol (HDL-C), and low-density lipoprotein cholesterol (LDL-C) between the two groups (all *p* > 0.05).

Liver function markers showed marked disparities. Both alanine aminotransferase (ALT) and aspartate aminotransferase (AST) levels were significantly higher among rural participants (ALT: 13.44 ± 5.86 vs. 9.19 ± 4.20 U/L, *p* = 0.004; AST: 22.04 ± 6.62 vs. 18.65 ± 3.75 U/L, *p* = 0.027). No significant difference was found in alkaline phosphatase (ALP) levels (*p* = 0.150). With respect to kidney function, creatinine and blood urea nitrogen (BUN) concentrations did not differ significantly between the rural and peri-urban groups (*p* = 0.226 and *p* = 0.996, respectively).

Among the serum electrolytes, a statistically significant difference was found in sodium (Na) levels, which were lower in the peri-urban group (135.32 ± 26.85 mmol/L) compared to the rural group (143.09 ± 1.79 mmol/L, *p* < 0.001). However, calcium, potassium, chloride, and carbon dioxide levels showed no significant differences across groups.

### 3.4. Vascular Inflammatory Biomarkers of Participants

**Figure 2:**
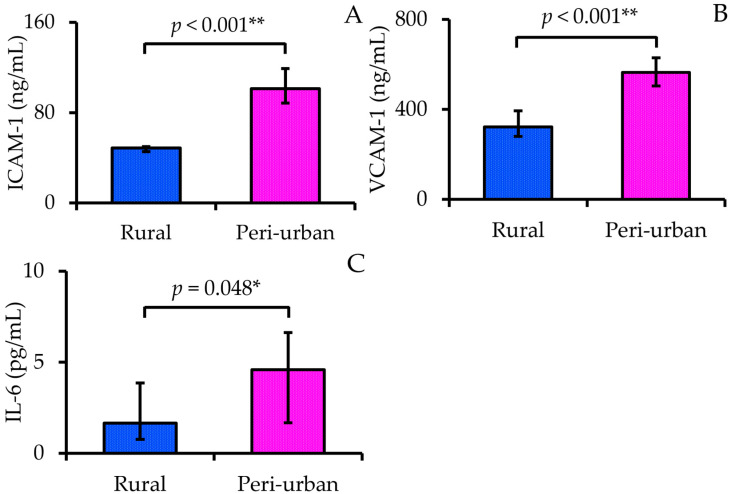
Serum concentrations of vascular inflammation markers—ICAM-1 (**A**), VCAM-1 (**B**), and IL-6 (**C**)—of participants residing in rural (n = 27) and peri-urban (n = 26) areas. Data are presented as median (Q1–Q3). Differences between the two areas were compared using the Mann–Whitney U test. Note: * *p* < 0.05; ** *p* < 0.01.

Serum concentrations of vascular inflammatory biomarkers differed significantly between participants residing in rural and peri-urban areas (Figure 2). Median ICAM-1 levels were markedly higher in the peri-urban group (101.26 [88.46–119.18] ng/mL) compared with the rural group (48.67 [45.43–49.82] ng/mL; *p* < 0.001). Similarly, VCAM-1 concentrations were significantly elevated among peri-urban participants (564.40 [504.40–628.40] ng/mL) relative to rural participants (321.50 [279.83–393.17] ng/mL; *p* < 0.001). In addition, IL-6 levels were higher in the peri-urban group (4.60 [1.68–6.63] pg/mL) than in the rural group (1.66 [0.76–3.85] pg/mL), with the difference reaching statistical significance (*p* = 0.048).

### 3.5. Energy and Nutrient Intakes of Participants

The distribution of energy intake derived from macronutrients—carbohydrates, protein, and fat—among participants residing in rural and peri-urban areas is illustrated in Table 5. No statistically significant differences were observed in the percent energy derived from macronutrients between rural and peri-urban participants. Median carbohydrate contribution was 54.65 (44.92–67.52)% vs. 59.39 (46.99–65.81)% (*p* = 0.957), protein 14.55 (12.43–21.97)% vs. 14.12 (11.75–20.91)% (*p* = 0.355), and fat 28.05 (13.64–34.71)% vs. 27.80 (19.88–34.06)% (*p* = 0.644).

Median (IQR) daily intakes are summarized in Table 6. Total energy and most macronutrients were comparable between rural and peri-urban participants, with no between-area differences for energy, carbohydrates, total protein, animal/vegetable protein, total fat, or dietary fiber (all *p* > 0.05). Peri-urban residents consumed more sugars [49.57 (26.20–86.80) vs. 27.09 (14.50–53.05) g; *p* = 0.016] and had markedly higher saturated fatty acid intake [19.04 (8.69–25.45) vs. 6.44 (2.21–12.35) g; *p* = 0.003]. In contrast, rural residents reported higher dietary cholesterol [470.49 (107.12–759.93) vs. 174.57 (90.86–302.19) mg; *p* = 0.018].

For micronutrients, rural participants had greater intakes of calcium [496.56 (278.99–728.75) vs. 314.33 (252.88–453.21) mg; *p* = 0.048], phosphorus [819.02 (678.84–1289.22) vs. 685.92 (516.69–989.95) mg; *p* = 0.013], potassium [1897.38 (1520.51–2619.09) vs. 1560.36 (1259.69–2167.93) mg; *p* = 0.040], selenium [55.54 (6.78–131.34) vs. 34.41 (24.96–53.31) µg; *p* = 0.004], and vitamin A as retinol activity equivalents [473.09 (288.40–811.75) vs. 212.29 (126.34–516.62) µg RAE; *p* = 0.024] and retinol [185.25 (6.71–297.65) vs. 87.84 (23.15–184.42) µg; *p* = 0.043], vitamin B2 [1.29 (0.85–1.63) vs. 0.98 (0.64–1.50) mg; *p* = 0.036], and vitamin C [70.23 (40.99–118.86) vs. 40.44 (16.81–65.88) mg; *p* = 0.017]. Sodium, iron, magnesium, copper, zinc, vitamin B1, vitamin B6, vitamin B12, niacin, and vitamin E were not significantly different (all *p* > 0.05).

### 3.6. Associations Between Nutrient Intakes and Vascular Inflammatory Biomarkers

Table 7 presents the results of multiple linear regression analyses examining the associations between specific nutrient intakes and circulating concentrations of ICAM-1, VCAM-1, and IL-6. All biomarkers and nutrient intakes were log-transformed, standardized β coefficients are reported, and models were adjusted for sex, age, physical activity, smoking status, alcohol consumption, BMI, WC, FBG, and total energy intake. Higher sugar intake was positively associated with all three biomarkers—ICAM-1 (*β* = 0.467, *p* < 0.001), VCAM-1 (*β* = 0.481, *p* < 0.001), and IL-6 (*β* = 0.557, *p* < 0.001). Total saturated fatty acids showed a positive association with ICAM-1 (*β* = 0.444, *p* = 0.002). Among antioxidant nutrients, selenium showed a significant inverse relationship with VCAM-1 (*β* = −0.406, *p* = 0.002), while higher vitamin A intake was associated with lower VCAM-1 levels (*β* = −0.447, *p* = 0.001). Calcium, phosphorus, iron, potassium, vitamin C, vitamin E, dietary fiber, and cholesterol were not significantly associated with any of the biomarkers in the adjusted models.

## 4. Discussion

This exploratory study investigated the associations between nutrient intake and vascular inflammatory biomarkers among apparently healthy adults residing in rural and peri-urban areas of Chiang Mai Province, Thailand, where seasonal PM2.5 pollution frequently exceeds the National Environment Board of Thailand-recommended thresholds. Participants from the two regions differed significantly in sociodemographic and behavioral characteristics, including sex, age, alcohol consumption, smoking, and physical activity levels.

Peri-urban participants were older than those in rural areas. Aging is a recognized non-modifiable risk factor for CVD, associated with increased oxidative stress, endothelial dysfunction, and elevated inflammatory biomarkers—even in asymptomatic individuals [24]. Conversely, the rural group had higher rates of alcohol consumption and smoking. Both behaviors are established modifiable risk factors for CVD, contributing to vascular inflammation through mechanisms involving oxidative stress and upregulation of cytokines and adhesion molecules [25,26,27].

Physical activity levels, as assessed by total MET-min/week, were significantly higher in the rural group than in the peri-urban group. This difference may reflect occupational patterns; nearly 44% of rural participants reported working in agriculture, a labor-intensive sector, whereas most peri-urban participants were employed in general, daily-wage labor (as 34.6% of peri-urban participants). Regular physical activity is known to confer substantial cardiovascular benefits, including improvements in endothelial function, reductions in inflammatory markers, and favorable modulation of lipid and glucose metabolism [28]. In a meta-analysis conducted by Wahid et al. (2016), individuals with higher activity levels had a 23% reduced risk of CVD mortality and a 17% lower risk of CVD incidence [29].

In addition to behavioral and lifestyle differences, this study observed significant variations in anthropometric and biochemical markers between rural and peri-urban participants. Individuals in peri-urban areas had significantly higher BMI than those in rural areas, with a trend toward higher WC. Both indicators are well-established predictors of CVD, reflecting central adiposity and metabolic risk [30]. The Asia-Pacific guidelines define BMI ≥ 23.0 kg/m^2^ and WC ≥ 90 cm (men) or ≥ 80 cm (women) as thresholds for elevated CVD risk in Asian populations [31].

Peri-urban participants also showed higher FBG levels, nearing the impaired fasting glucose range (100–125 mg/dL) per ADA criteria [32]. Although still within normal limits, this may signal emerging insulin resistance, possibly due to higher sugar intake, greater adiposity, and reduced physical activity. Prior research indicates that even high-normal FBG is linked to vascular inflammation and CVD risk [33]. Interestingly, rural participants had significantly higher AST and ALT levels, though within normal limits. This may reflect greater alcohol intake (74.1%) or hepatic stress [34]. Additionally, serum sodium concentration was also significantly higher among rural participants compared to those in the peri-urban area, though both were within the physiological range (typically 135–146 mmol/L) [35]. The higher sodium levels in rural participants may be attributed to greater physical exertion and insensible fluid loss through perspiration due to agricultural labor or variations in dietary salt intake. It is important to note that even subtle differences in serum sodium levels can affect vascular tone and blood pressure regulation, particularly in populations at risk of cardiovascular disease [36].

The present study revealed significantly elevated serum concentrations of all three vascular inflammatory biomarkers—ICAM-1, VCAM-1, and IL-6—among participants residing in peri-urban areas compared to those in rural areas. These findings suggest a heightened state of subclinical vascular inflammation in the peri-urban population, potentially linked to lifestyle factors, dietary habits, and chronic environmental exposures such as PM2.5. IL-6 is a key pro-inflammatory cytokine that plays a central role in the progression of atherosclerosis by promoting hepatic synthesis of acute-phase reactants, activating endothelial cells, and facilitating leukocyte recruitment into vascular tissue [5,6]. Similarly, ICAM-1 and VCAM-1 are adhesion molecules upregulated in response to inflammatory stimuli and oxidative stress; their expression on endothelial surfaces mediates leukocyte adhesion and transmigration, which are crucial early events in vascular injury and plaque development [7]. Elevated levels of these biomarkers are indicative of endothelial activation and are associated with increased CVD risk [5,6,7]. The higher inflammatory marker levels observed in peri-urban residents may reflect an interplay between lower physical activity, higher adiposity, greater intake of pro-inflammatory nutrients (e.g., sugars, saturated fats), and increased exposure to environmental stressors.

The current study identified notable differences in dietary intake patterns between rural and peri-urban participants, which may partly explain the observed disparities in vascular inflammatory biomarkers. Specifically, participants from peri-urban areas exhibited significantly higher intakes of sugars and saturated fatty acids, whereas those residing in rural areas consumed greater amounts of dietary fiber, cholesterol, antioxidant vitamins (e.g., vitamins A, C, and E), and several essential minerals, including calcium, phosphorus, iron, potassium, and selenium.

These differences likely reflect broader lifestyle and food environment variations between the two settings. Rural participants, primarily engaged in subsistence agriculture, demonstrated dietary behaviors consistent with traditional Northern Thai eating patterns. Their diets frequently included locally grown vegetables—such as Chinese cabbage (*Brassica rapa*), white cabbage, and kale (*Brassica oleracea*)—which are often consumed boiled, stir-fried, or as accompaniments to chili pastes (nam phrik). A particularly common dish is “jor pak kad,” a regional vegetable stew made from Chinese mustard greens, pork belly, and local seasonings. In addition, the habitual consumption of eggs—often 2–3 eggs per day in some individuals—was observed, reflecting an accessible and nutrient-dense protein source in rural settings. These dietary habits align with the higher intakes of cholesterol, vitamin A precursors, vitamin E, and minerals such as selenium and iron observed in this group. For example, leafy greens like kale and Chinese mustard greens are rich in provitamin A, calcium, and iron [37,38], while pork and eggs contribute significant amounts of cholesterol, selenium, and phosphorus [39,40,41]. Furthermore, the most commonly consumed fruit among rural participants was the Namwa banana (*Musa sapientum*), which is naturally abundant in dietary fiber, potassium, vitamin C, and bioactive compounds such as dopamine [42]. These nutrients are known to support cardiovascular health, regulate blood pressure, and mitigate oxidative stress [42,43,44]—further complementing the nutrient profile derived from traditional dietary patterns in this population.

In contrast, peri-urban participants—who generally have greater access to food outlets and processed food products—tended to consume a more Westernized diet characterized by a higher intake of energy-dense and nutrient-poor foods. Dietary recall interviews indicated frequent consumption of fried foods, processed meats, and sugar-sweetened beverages, including sweetened coffee drinks, milk teas, and carbonated soft drinks. These foods are known to be high in added sugars and saturated fats, which is consistent with the significantly greater intake of these components among peri-urban participants in our study. Prior research has shown that diets high in saturated fats and refined sugars are associated with elevated inflammatory responses, oxidative stress, and endothelial dysfunction [17,18].

Regression analyses—adjusted for sex, age, physical activity, smoking, alcohol use, BMI, WC, FBG, and total energy intake—revealed a robust positive relationship between sugar intake and all three inflammatory markers. This concurs with prior studies indicating that excessive added sugars, especially from sugar-sweetened beverages, promote systemic inflammation via increased free fatty acid mobilization, activation of NF-κB pathways, and upregulation of pro-inflammatory cytokines, including IL-6 and adhesion molecules [45,46,47].

Conversely, several micronutrients demonstrated protective associations. Dietary selenium intake showed a particularly strong negative association with VCAM-1. Selenium is a key micronutrient involved in redox regulation and immune modulation. Higher selenium intake has been associated with reduced cardiovascular and inflammatory risk in large cohort studies, including the American National Health and Nutrition Examination Survey (NHANES) analyses demonstrating a nonlinear inverse association with CVD prevalence and mortality [48]. Among antioxidant nutrients, vitamin A intake exhibited a significant inverse association with VCAM-1, corroborating research underscoring its role in reducing endothelial activation and adhesion molecule expression via antioxidative and anti-inflammatory mechanisms [49,50]. Vitamin A–deficient rodents displayed elevated vascular oxidative stress and increased endothelial expression of VCAM-1, indicating that sufficient vitamin A status may help maintain endothelial integrity by attenuating oxidative and inflammatory pathways linked to adhesion molecule upregulation [51]. While there are few direct mechanistic studies that specifically link vitamin A to VCAM-1 modulation, a broader body of literature supports the concept that antioxidant vitamins—particularly fat-soluble ones like vitamin A and carotenoids—help preserve endothelial function by neutralizing ROS, curbing adhesion molecule expression, and countering vascular inflammation [49,50,51,52].

Despite distinct dietary patterns between rural and peri-urban participants, both groups consumed excessive sodium—averaging over 3000 mg/day—far above the Thai Ministry of Public Health’s recommended limit of 2000 mg/day for adults to reduce hypertension and cardiovascular risk [53]. High sodium intake is a modifiable risk factor linked to elevated blood pressure, endothelial dysfunction, and increased cardiovascular morbidity. Meta-analyses have demonstrated a strong dose–response effect, showing that reducing sodium significantly lowers blood pressure, especially in hypertensive individuals [54,55]. In Thailand, persistently high sodium intake is reported across all age groups, with major contributors including fish sauce, soy sauce, and other salty condiments and processed foods [56]. This study’s findings underscore the urgent need for targeted interventions—such as public nutrition education, product reformulation, and policy measures like front-of-pack labeling—to lower sodium consumption. Tailored approaches are essential for both rural and peri-urban populations, who may differ in dietary access but face similar sodium-related health risks. Such strategies are especially important in communities exposed to additional cardiovascular stressors such as PM2.5 air pollution.

This study has certain limitations. As an exploratory cross-sectional design with a relatively small sample size, the findings should be interpreted with caution. Nonetheless, these preliminary results provide an important foundation for future investigations with larger and more diverse populations and broader research scopes. Another limitation is that while the study focused on vascular inflammatory markers, it did not assess other biological pathways potentially affected by PM2.5 exposure. Thus, the underlying mechanisms could not be fully elucidated. Future studies should therefore incorporate additional biomarkers—such as oxidative stress indicators (e.g., malondialdehyde, 8-isoprostane), endothelial function markers (e.g., nitric oxide, endothelin-1), and epigenetic modifications—to better clarify the biological links between chronic PM2.5 exposure, diet, and cardiovascular health. Despite these limitations, this exploratory study yields valuable insights. The findings highlight that nutrient-rich dietary patterns, particularly those characterized by higher intakes of antioxidants, dietary fiber, and essential micronutrients, may mitigate vascular inflammation, while more Westernized dietary patterns may increase cardiovascular risk in peri-urban settings. These insights not only strengthen the scientific rationale for integrating nutrition into environmental health research but also offer practical applications for community-based interventions and public health strategies aimed at reducing the combined burden of poor diet and air pollution.

## 5. Conclusions

This exploratory study provides preliminary evidence that nutrient intake may modulate vascular inflammatory responses among adults exposed to PM2.5 pollution in Chiang Mai, Thailand. Participants residing in peri-urban areas exhibited higher levels of vascular inflammation biomarkers (ICAM-1, VCAM-1, and IL-6) compared with those living in rural areas, which may be attributable to their higher sugar consumption. Conversely, rural participants reported greater intakes of selenium and vitamin A, both of which showed inverse associations with VCAM-1 levels. These findings suggest that traditional rural dietary patterns may confer protective effects against vascular inflammation, even in populations exposed to environmental stressors. Future studies with larger sample sizes and longitudinal designs are warranted to confirm and further elucidate these associations.

## Figures and Tables

**Table 1:** The average annual concentrations of particulate matter 2.5 recorded between 2021 and 2023 in Omkoi (rural) and San Pa Tong (peri-urban) districts.

Districts	Annual Average Particulate Matter 2.5 Concentration (2021–2023) (µg/m^3^) ^ns^
Omkoi District	15.58 ± 5.60
San Pa Tong District	19.27 ± 7.32

Data are presented as mean ± standard deviation. Note: ^ns^ indicates no significant difference between the two areas.

**Table 2:** Sociodemographic and behavioral characteristics of participants residing in rural and peri-urban areas.

Characteristics	Rural (n = 27)	Peri-Urban (n = 26)	Total (n = 53)	*p*
**Sex**				<0.001 **
Male	13 (48.15%)	1 (3.85%)	14 (26.42%)	
Female	14 (51.85%)	25 (96.15%)	39 (73.58%)	
**Age (years)**	50.04 ± 7.58	56.04 ± 7.12	52.92 ± 7.89	0.005 **
**Alcohol consumption**				0.009 **
Never drank	7 (25.93%)	16 (61.54%)	23 (43.40%)	
Drank	20 (74.07%)	10 (38.46%)	30 (56.60%)	
**Smoking status**				0.002 **
Never smoked	16 (59.26%)	23 (88.46%)	39 (73.58%)	
Current smoker	9 (33.33%)	0	9 (17.98%)	
Former smoker	2 (7.41%)	3 (11.54%)	5 (9.43%)	
**Total physical activity** **(MET-min/week)** ^1^	9492.00 (2772.00–17,598.00)	2451.00 (1339.50–8671.50)	6612.00 (1812.00–11,838.00)	0.020 *
Vigorous activity (MET-min/week) ^1^	3360.00 (0.00–13,440.00)	720.00 (0.00–4800.00)	1200.00 (0.00–6720.00)	0.082
Moderate activity (MET-min/week) ^1^	480.00 (0.00–720.00)	1320.00 (360.00–1680.00)	720.00 (0.00–1680.00)	0.001 **
Walking (MET-min/week) ^1^	1584.00 (594.00–4158.00)	297 (0.00–1386.00)	1386.00 (132.00–2772.00)	0.019 *

Data are expressed as number and percentage for categorical variables or as mean ± standard deviation for continuous variables. ^1^ Physical activity variables, which were non-normally distributed, are presented as median (Q1–Q3), and group comparisons were performed using the Mann–Whitney U test. Note: * indicates a statistically significant difference at *p* < 0.05, and ** indicates a statistically significant difference at *p* < 0.01. MET, metabolic equivalent of task.

**Table 3:** Physical examination parameters of participants residing in rural and peri-urban areas.

Parameters	Rural (n = 27)	Peri-Urban (n = 26)	*p*
Body height (cm)	158.27 ± 9.80	153.68 ± 6.66	0.082
Body weight (kg)	58.32 ± 12.04	61.00 ± 10.93	0.464
Body mass index (kg/m^2^)	23.33 ± 4.92	25.76 ± 3.76	0.004 **
Waist circumference (cm)	79.94 ± 12.22	84.17 ± 9.54	0.058
Male	80.77 ± 7.42	93.50	0.171
Female	79.04 ± 15.7	83.76 ± 9.55	0.056
Waist-to-hip ratio	0.85 ± 0.07	0.85 ± 0.06	0.756
Male	0.87 ± 0.06	0.93	0.385
Female	0.84 ± 0.07	0.84 ± 0.05	0.570
Systolic blood pressure (mmHg)	120.81 ± 8.47	121.23 ± 10.82	0.877
Diastolic blood pressure (mmHg)	75.37 ± 8.99	75.23 ± 8.74	0.955
Heart rate (bpm)	74.93 ± 14.33	76.08 ± 8.36	0.790

Data are expressed as mean ± standard deviation. Differences between groups were assessed using the independent *t*-test. Note: ** indicates a statistically significant difference at *p* < 0.01.

**Table 4:** Clinical biochemistry parameters of participants residing in rural and peri-urban areas.

Parameters	Rural (n = 27)	Peri-Urban (n = 26)	*p*
Fasting blood glucose (mg/dL)	97.19 ± 18.59	100.85 ± 14.03	0.050 *
**Lipid profiles**			
Total cholesterol (mg/dL)	202.81 ± 41.93	194.38 ± 43.51	0.476
Triglyceride (mg/dL)	109.41 ± 58.59	103.88 ± 39.94	0.972
High-density lipoprotein cholesterol (mg/dL)	44.45 ± 8.44	42.80 ± 11.79	0.559
Low-density lipoprotein cholesterol (mg/dL)	150.59 ± 48.64	142.23 ± 53.50	0.554
**Liver function tests**			
Alanine aminotransferase (U/L)	13.44 ± 5.86	9.19 ± 4.20	0.004 **
Aspartate aminotransferase (U/L)	22.04 ± 6.62	18.65 ± 3.75	0.027 *
Alkaline phosphatase (U/L)	101.56 ± 24.53	91.38 ± 26.22	0.150
**Kidney function tests**			
Creatinine (mg/dL)	0.80 ± 0.41	0.67 ± 0.27	0.226
Blood urea nitrogen (mg/dL)	12.25 ± 5.08	12.24 ± 2.99	0.996
**Serum electrolytes**			
Calcium (mg/dL)	9.07 ± 0.34	8.90 ± 0.31	0.067
Sodium (mmol/L)	143.09 ± 1.79	135.32 ± 26.85	0.007 **
Potassium (mmol/L)	4.02 ± 0.44	4.04 ± 0.29	0.857
Chloride (mmol/L)	106.77 ± 1.90	106.21 ± 2.15	0.317
Carbon dioxide (mmol/L)	24.56 ± 2.99	25.45 ± 1.66	0.188

Data are expressed as mean ± standard deviation. Differences between groups were assessed using the independent *t*-test. Note: * indicates a statistically significant difference at *p* ≤ 0.05, and ** indicates a statistically significant difference at *p* < 0.01.

**Table 5:** Percent energy intake from macronutrients between rural and peri-urban groups.

Macronutrients	Rural (n = 27)	Peri-Urban (n = 26)	*p* ^ns^
Carbohydrate (%)	54.65 (44.92–67.52)	59.39 (46.99–65.81)	0.957
Protein (%)	14.55 (12.43–21.97)	14.12 (11.75–20.91)	0.355
Fat (%)	28.05 (13.64–34.71)	27.80 (19.88–34.06)	0.644

Data are presented as median (Q1–Q3). Differences between the two areas were compared using the Mann–Whitney U test. Note: ^ns^ indicates no statistically significant difference between groups.

**Table 6:** Daily intake of energy and nutrients among participants in rural and peri-urban areas.

Variables	Rural (n = 27)	Peri-Urban (n = 26)	*p*
Energy (kcal)	1734.12 (1394.09–2058.34)	1808.27 (1368.30–2107.04)	0.735
Carbohydrates (g)	234.13 (190.15–272.34)	231.77 (204.40–280.56)	0.510
Sugars (g)	27.09 (14.50–53.05)	49.57 (26.20–68.60)	0.030 *
Proteins (g)	64.34 (52.86–93.75)	57.37 (46.93–85.56)	0.594
Animal protein (g)	38.28 (21.96–59.42)	33.09 (16.34–53.69)	0.702
Vegetable protein (g)	18.85 (15.90–23.70)	18.02 (15.55–20.56)	0.270
Fats (g)	48.67 (31.85–82.09)	50.34 (32.06–80.59)	0.557
Total saturated fatty acids (g)	6.44 (2.21–12.35)	19.04 (8.69–25.45)	0.002 **
Cholesterol (mg)	470.49 (107.12–759.93)	174.57 (90.86–302.19)	0.013 *
Calcium (mg)	496.56 (278.99–728.75)	314.33 (252.88–453.21)	0.048 *
Phosphorus (mg)	819.02 (678.84–1289.22)	685.92 (516.69–819.95)	0.031 *
Iron (mg)	13.05 (10.49–21.40)	10.27 (7.97–16.27)	0.046 *
Potassium (mg)	1897.38 (1520.51–2619.09)	1560.36 (1259.69–2167.93)	0.039 *
Sodium (mg)	3174.42 (2578.65–4108.96)	2944.25 (2085.47–3999.52)	0.302
Magnesium (mg)	47.05 (26.28–100.62)	34.30 (19.61–58.14)	0.130
Copper (mg)	0.84 (0.65–1.30)	0.75 (0.61–1.09)	0.294
Selenium (μg)	55.54 (6.78–131.34)	34.41 (24.96–53.31)	0.045 *
Zinc (mg)	6.24 (4.32–7.10)	4.70 (3.70–5.69)	0.055
Vitamin A (μg RAE)	473.09 (288.40–811.75)	212.29 (126.34–513.62)	0.024 *
Retinol (μg)	185.25 (6.71–297.65)	87.84 (23.15–184.42)	0.824
β-carotene (μg)	1575.86 (362.14–6680.85)	1076.78 (392.89–2312.90)	0.160
Vitamin B1 (mg)	0.87 (0.51–1.46)	1.33 (0.77–2.54)	0.091
Vitamin B2 (mg)	1.29 (0.85–1.63)	1.08 (0.64–1.50)	0.328
Vitamin B6 (mg)	0.56 (0.06–1.04)	0.48 (0.23–0.86)	0.790
Vitamin B12 (mg)	1.82 (0.00–3.17)	0.46 (0.00–2.20)	0.688
Niacin (mg)	14.99 (12.48–22.36)	15.17 (11.78–19.01)	0.581
Vitamin C (mg)	70.23 (40.99–118.86)	40.44 (16.81–68.81)	0.017 *
Vitamin E (mg)	1.77 (0.21–4.89)	0.42 (0.25–0.72)	0.027 *
Dietary fiber (g)	13.06 (8.47–17.96)	7.25 (5.53–16.51)	0.052

Data are presented as median (Q1–Q3). Differences between the two areas were compared using the Mann–Whitney U test. Note: * *p* < 0.05; ** *p* < 0.01.

**Table 7:** Linear regression analysis of the associations between nutrient intakes and circulating concentrations of vascular inflammatory biomarkers.

Nutrients	ICAM-1		VCAM-1		IL-6	
	*β*	*p*	*β*	*p*	*β*	*p*
Sugars	0.467	**<0.001 ^3^**	0.481	**<0.001 ^2^**	0.557	**<0.001 ^1^**
Total saturated fatty acids	0.444	**0.002 ^5^**	0.212	0.148	0.127	0.726
Cholesterol	−0.119	0.363	−0.096	0.514	−0.055	0.857
Calcium	−0.171	0.180	−0.043	0.755	−0.319	0.287
Phosphorus	−0.129	0.439	−0.101	0.537	0.096	0.621
Iron	−0.221	0.131	−0.078	0.579	0.132	0.594
Potassium	−0.352	0.014	−0.281	0.053	0.419	0.178
Selenium	−0.097	0.150	−0.406	**0.002 ^6^**	−0.218	0.319
Vitamin A	−0.262	0.072	−0.447	**0.001 ^4^**	−0.223	0.417
Vitamin C	−0.293	0.014	−0.187	0.204	−0.271	0.319
Vitamin E	−0.136	0.256	−0.231	0.097	−0.339	0.266
Dietary fiber	−0.246	0.045	−0.332	0.010	−0.453	0.074

Data are expressed as standardized regression coefficients (β) and corresponding *p*-values from multiple linear regression models (n = 53). All inflammatory biomarkers and nutrient intakes were log-transformed prior to analysis. All models were adjusted for sex, age, physical activity, smoking status, alcohol consumption, BMI, WC, FBG, and total energy intake. Multiple testing was controlled using the Benjamini–Hochberg procedure across 36 comparisons; superscript numerals indicate the rank-specific significance thresholds: **^1^** *p* < 0.0014, **^2^** *p* < 0.0028, **^3^** *p* < 0.0042, **^4^** *p* < 0.0056, **^5^** *p* < 0.0069, and **^6^** *p* < 0.0083. ICAM-1, intercellular adhesion molecule-1; VCAM-1, vascular cell adhesion molecule-1; IL-6, interleukin-6.

## Data Availability

The data presented in this study are available upon request from the corresponding author.
